# Training medical students on rare disorders

**DOI:** 10.1186/1750-1172-7-S2-A15

**Published:** 2012-11-22

**Authors:** Paula C Byrne

**Affiliations:** 1 School of Medicine and Medical Science, University College Dublin, Ireland

## 

A significant challenge faced by families and patients affected by rare diseases is the general lack of awareness among medical professionals of the sheer number of rare diseases that exist and the sometimes devastating impact a rare disease can have on all aspects of a person’s life. Many doctors believe that they are unlikely to come across a rare disease in their professional careers and simply do not realise that *it is not rare to have a rare disease*. The School of Medicine and Medical Science in University College Dublin has developed an innovative educational module that aims to increase awareness of rare diseases among medical students.

We have developed an elective module with contributions from patient organisations, clinicians, academic staff, research scientists and pharmaceutical industry. The module is offered to undergraduate medical students in the final year of their pre-clinical training. The module was designated a grade neutral module which makes no impact on the student’s overall grade that academic year. The method of assessment had to reflect the overall aim of the module, which was to increase awareness of rare diseases among medical students and not examine the level of scientific knowledge of a host of rare disorders. Assessment of the module had two distinct components. The first was a reflective learning journal that had to be completed by the student at regular intervals during the semester. Students also had to prepare an information pamphlet suitable for a medical professional detailing genetic basis, symptoms and treatment of the disorder chosen, as well as resources for further information.

Feedback from the module has been extremely positive. All contributors were very enthusiastic about the module and very keen to be involved. Feedback from the students was exceptionally complimentary. Many students commended the multidisciplinary approach taken. The highlights of the module were the talks given either directly by the patients themselves, or by representatives of patient support organisations. These accounted for approximately 30% of the lectures. There is no doubt that these sessions proved both inspirational and memorable for the students. Entries to the reflective journal described how the module and in particular the experiences described by the patients prompted many students to research particular disorders in more depth and made a lasting impact on them. The feedback from the module has been overwhelmingly positive and shows the advantage of using a multidisciplinary approach where the student can hear directly from the patient, learn about the importance of research and advances in treatment of rare disorders and identify resources that will be useful in their future professional careers.

